# Supplementary material for: Enhancing Mezcal Production Efficiency by Adding an Inoculant of Native Saccharomyces cerevisiae to a Standardized Fermentation Must
Source: Foods. 2025 Jan 21;14(3):341. doi: 10.3390/foods14030341 (PMC11817156; doi:10.3390/foods14030341)
Supplement: Supplementary file 1 [file foods-14-00341-s001.zip › Figure S1.pdf]

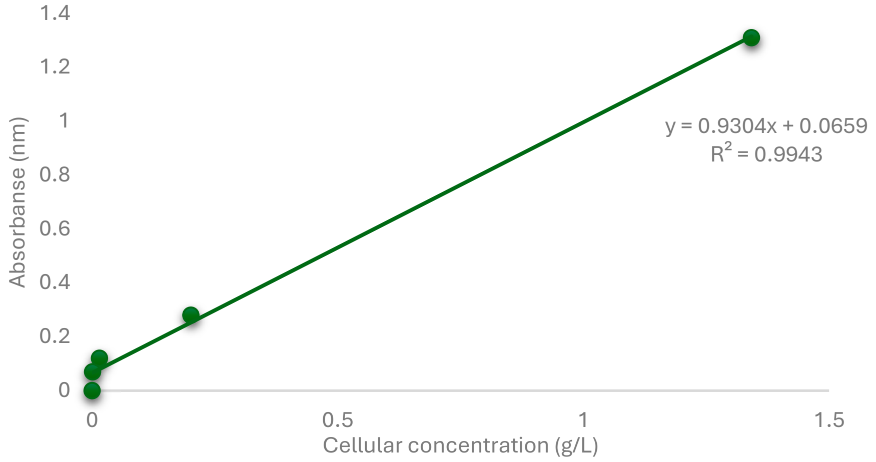

Figure S1. Calibration curve of absorbance and cellular concentration by dry weight of Saccharomyces cerevisiae ITD-00185.
