# Supplementary material for: Enhancing Mezcal Production Efficiency by Adding an Inoculant of Native Saccharomyces cerevisiae to a Standardized Fermentation Must
Source: Foods. 2025 Jan 21;14(3):341. doi: 10.3390/foods14030341 (PMC11817156; doi:10.3390/foods14030341)
Supplement: Supplementary file 1 [file foods-14-00341-s001.zip › Table S1.pdf]

Table S1. Analyzed fermentation parameters of CDC in inoculated fermentations ($10X^6$ Cell/mL) de agave a 48h de fermentación. of agave at 48 h of fermentation. Maximum specific growth rate (μ_{max}), substrate consumption in percentage (S), ethanol concentration produced (P), yield ($Y_{P/S}$), y and productivity (Q_P).

Kinetics	Conditions		μ_{Max} (h^{-1})	S (%)	P (g/L)	$Y_{P/S}$	Q_p (g/Lh)
	Substrate (g/L)	YAN (g/L)					
1	90	0.168	0.21 ±0.0321 ^{A}	91.18 ±0.07^{AB}	41.45±1.73 ^{BC}	0.49±0.02^{A}	1.29±0.02^{D}
2	120	0.084	0.12±0.0068 ^{DEF}	90.98±0.27 ^{B}	49.47±1.52 ^{AB}	0.44±0.01^{B}	1.25±0.00^{D}
3	98.7	0.108	0.14±0.0046 ^{BC}	90.67±1.35 ^{B}	39.14±5.80 ^{C}	0.44±0.08^{B}	1.22±0.00^{DE}
4	141.2	0.108	0.12±0.0025 ^{DEF}	91.74±0.00 ^{AB}	52.86±0.13 ^{A}	0.42±0.00^{B}	1.16±0.03^{E}
5	120	0.168	0.13±0.0031 ^{BCD}	91.80±0.36 ^{AB}	53.15±3.96 ^{A}	0.46±0.03^{AB}	1.53±0.02^{A}
6	150	0.168	0.10±0.0038 ^{F}	92.9±0.30 ^{A}	54.76±1.12 ^{A}	0.40±0.01^{B}	1.43±0.04^{BC}
7	120	0.252	0.12±0.0033 ^{CDE}	91.94±0.02 ^{AB}	42.64±1.25 ^{BC}	0.41±0.01^{B}	1.54±0.01^{A}
8	141.2	0.227	0.10±0.0014 ^{EF}	92.68±0.51 ^{A}	53.38±0.76 ^{A}	0.41±0.01^{B}	1.42±0.00^{C}
9	120	0.168	0.12±0.0019 ^{DE}	92.27±0.20 ^{AB}	51.14±1.76 ^{A}	0.47±0.00^{AB}	1.52±0.02^{A}
10	98.7	0.227	0.15±0.0029 ^{B}	91.76±0.47 ^{AB}	38.49±0.97 ^{C}	0.43±0.02^{B}	1.31±0.04^{D}
11	120	0.168	0.13±0.0031 ^{BCD}	91.88±0.09 ^{AB}	47.53±1.24 ^{AB}	0.45±0.01^{AB}	1.52±0.02^{AB}
