# Supplementary material for: Enhancing Mezcal Production Efficiency by Adding an Inoculant of Native Saccharomyces cerevisiae to a Standardized Fermentation Must
Source: Foods. 2025 Jan 21;14(3):341. doi: 10.3390/foods14030341 (PMC11817156; doi:10.3390/foods14030341)
Supplement: Supplementary file 1 [file foods-14-00341-s001.zip › Table S2.pdf]

Table S2. Relative abundance in percentage of strains identified by MALDI-TOF from CDC fermentations in agave juice at 0, 24 and 48 hours of fermentation

	**CDC condition**																																
**Microorganism**	**1**			**2**			**3**			**4**			**5**			**6**			**7**			**8**			**9**			**10**			**11**		
	**0**	**24**	**48**	**0**	**24**	**48**	**0**	**24**	**48**	**0**	**24**	**48**	**0**	**24**	**48**	**0**	**24**	**48**	**0**	**24**	**48**	**0**	**24**	**48**	**0**	**24**	**48**	**0**	**24**	**48**	**0**	**24**	**48**
Saccharomyces cerevisiae	0	100	89	94	100	100	100	100	100	100	100	100	100	100	100	100	0	100	17	100	80	0	100	100	0	100	100	80	100	100	67	100	100
Acetobacter cerevisiae	0	0	4	0	0	0	0	0	0	0	0	0	0	0	0	0	0	0	0	0	0	0	0	0	0	0	0	0	0	0	0	0	0
Gluconobacter oxydans	0	0	7	6	0	0	0	0	0	0	0	0	0	0	0	0	0	0	0	0	20	0	0	0	0	0	0	0	0	0	0	0	0
Acinetobacter radioresistens	0	0	0	0	0	0	0	0	0	0	0	0	0	0	0	0	0	0	17	0	0	0	0	0	0	0	0	0	0	0	0	0	0
Brevibacillus centrosporus	0	0	0	0	0	0	0	0	0	0	0	0	0	0	0	0	0	0	66	0	0	0	0	0	0	0	0	0	0	0	0	0	0
Bacillus subtilis	0	0	0	0	0	0	0	0	0	0	0	0	0	0	0	0	0	0	0	0	0	0	0	0	0	0	0	20	0	0	33	0	0
