# Supplementary material for: Enhancing Mezcal Production Efficiency by Adding an Inoculant of Native Saccharomyces cerevisiae to a Standardized Fermentation Must
Source: Foods. 2025 Jan 21;14(3):341. doi: 10.3390/foods14030341 (PMC11817156; doi:10.3390/foods14030341)
Supplement: Supplementary file 1 [file foods-14-00341-s001.zip › Table S3.pdf]

Table S3. Determination of agave bagasse fermentation parameters at the pilot plant level at 48 h (C1) and 120 h (C2 and C3). AR_0 (concentration of reducing sugars at 0 h), ARf (concentration of reducing sugars at time to end of fermentation), Consumption (Percentage of consumption of reducing sugars at time to end of fermentation), Mezcal (Volume of Mezcal obtained at the end of fermentation), Y kg pine/ L mezcal (Yield of obtaining Mezcal per kilogram of pine used), NA (not applicable)

No.	Conditions			Fermentation time (h)	AR_o (g/L)	AR_f (g/L)	$Consumption_{AR}$ (%)	Mezcal (L)	$Y_{kg\ agave/\ L\ mezcal}$
	substrate (g/L)	YAN (g/L)	Inoculum (Cel/mL)						
C1	120	0.227	$1x10^6$	48	118.67±7.94	21.23±3.65	82.10±3.07	0.36	11.61
C2	120	0.227	NA	120	110.85±5.52	5.80±0.02	94.76±0.24	0.30	13.93
C3	120	NA	NA	120	113.53±5.64	73.95±0.10	34.78±3.15	0.11	38
